# Is extra virgin olive oil a promising remedy for reducing the impact of postmenopausal osteoporosis? An experimental study

**DOI:** 10.3389/fvets.2025.1555779

**Published:** 2025-03-07

**Authors:** El-Sayed El-Shafaey, Eman Ali, Magda Elkomy, Mohamed Abdo Rizk, Saleh Altuwaijri, Saleh Albarrak

**Affiliations:** ^1^Department of Surgery, Anesthesiology and Radiology, Faculty of Veterinary Medicine, Mansoura University, Mansoura, Dakahlia, Egypt; ^2^Department of Veterinary Surgery, Salam Veterinary Group, Buraydah, Qassim, Saudi Arabia; ^3^Department of Zoology, Faculty of Science, Mansoura University, Mansoura, Dakahlia, Egypt; ^4^Department of Internal Medicine, Faculty of Veterinary Medicine, Mansoura University, Mansoura, Dakahlia, Egypt; ^5^Department of Pathology and Laboratory Diagnosis, College of Veterinary Medicine, Qassim University, Buraydah, Saudi Arabia

**Keywords:** olive oil, osteoporosis, ovariectomy, postmenopausal, rats, tumor necrosis factor-α

## Abstract

**Introduction:**

Osteoporosis, particularly postmenopausal osteoporosis, is a significant global health challenge with limited treatment options due to severe side effects associated with the long-term use of conventional therapies. Therefore, this study aims to provide a potentially novel therapeutic approach by examining olive oil's effects on bone mineral density (BMD), biochemical markers, biomechanical properties, and histopathological changes in an ovariectomized (OVX) rat model.

**Methods:**

In this study, Twenty-four 6-month-old female Wistar rats were randomly allocated into four equal groups (*n* = 6 rats, for each group): control group, rats given 1mL/100g olive oil, ovariectomized rats (OVX-group), and OVX rats treated with olive oil. The femoral bone mineral density (BMD), biochemical parameters, biomechanical properties, and histopathological features were studied.

**Results:**

After 3 months of extra virgin olive oil treatment, there were significant improvements in the different estimated parameters. This was demonstrated by preventing the changes in bone remodeling and BMD, improving the hormonal changes, oxidant/antioxidant imbalance, and abnormal levels of pro-inflammatory cytokines associated with OVX-induced osteoporosis. In addition, there was a marked improvement in the histological architecture of the cancellous and cortical bone appearance.

**Conclusion:**

Olive oil dietary intake effectively reduces the impact of osteoporosis induced by ovariectomy in rats, suggesting a potentially feasible treatment option for postmenopausal osteoporosis that benefits bone architecture without any detrimental side effects on women's health.

## Introduction

Osteoporosis is a common metabolic bone disease characterized by low bone mineral density (BMD) and deterioration of bone microarchitecture. Various causes of osteoporosis exist, but the most prevalent one is bone loss, which is linked to a lack of estrogen that occurs after menopause (1).

Osteoporotic fractures lead to significant morbidity, mortality, and healthcare costs. It is estimated that osteoporosis can cause ~9 million fractures annually and affect around 200 million individuals worldwide, with 34% of women over the age of 50 being affected. Therefore, the prevention and treatment of osteoporosis are crucial to avoid osteoporotic fractures (2). However, many pharmacotherapies have been explored as potential treatments for postmenopausal osteoporosis, not all patients exhibit a positive response to these synthetic medications, and some even experience severe side effects (1). Hence, the search for alternative agents that are both effective and safe for preventing and treating osteoporosis in postmenopausal women remains a significant concern (3).

Many studies have revealed insights into the search for natural compounds that possess anti-osteoporotic properties and cause minimal side effects. Medicinal plants offer safe alternatives to current therapies. Olive oil is a crucial element of the Mediterranean diet, which is associated with various health benefits (4). Research has demonstrated that olive oil can enhance different aspects of human health and has protective effects against heart disease, cancer, neurodegeneration, diabetes, and aging. Importantly, olive oil facilitates bone mineralization and development and may contribute to maintaining bone density. This is achieved through mechanisms involving increased bone formation, inhibition of bone resorption, and potentially reducing oxidative stress and inflammation (5).

Indeed, the Ovariectomy model is widely employed for investigating phenomena related to postmenopausal osteoporosis. It has been firmly recorded that ovariectomy induces the depletion of bone mass and an elevation in bone turnover in rats. Therefore, rat models of osteoporosis that undergo ovariectomy can effectively replicate conditions in postmenopausal women and are appropriate for evaluating potential treatments aimed at preventing or treating osteoporosis (6). Therefore, the present study was designed to:

- Evaluate the effects of extra virgin olive oil on bone mineral density (BMD) in ovariectomized rats.
- Analyze biochemical parameters and histological bone structure changes induced by olive oil supplementation.

## Materials and methods

### Animals

Twenty-four female Wistar rats, weighing 160 ± 10 g, were used in this experiment. The rats were obtained from the animal house of the Biological Products and Vaccines (VACSERA, Cairo, Egypt). They were housed in sterilized stainless-steel cages under controlled environmental conditions of 25°C and a 12-h light/dark cycle. The rats were allowed to acclimate for 1 week before the start of the experiment and were supplied with a standard diet and unlimited access to water.

### Study design

The osteoporosis model was induced by performing bilateral ovariectomy, following the standard established protocols according to Yousefzadeh et al. (6). Subsequently, the experimental animals were divided into four equitably sized groups, each comprising six rats. Group I served as the control group, consisting of healthy rats supplied with the standard diet without additional treatment throughout the study duration. Group II, the olive oil group, involved healthy rats that received 1 mL/100 g an orally administered supplement of extra virgin olive oil (Sigma-Aldrich, St. Louis, MO, USA; fatty acids composition is: 75% monounsaturates, 13% polyunsaturates and 12% saturates, and phenolic concentration is 200 mg/100 g olive oil). Group III, the OVX group, comprised of OVX rats that received a standard diet without any additional treatment. Lastly, Group IV, the OVX + olive oil group included OVX rats that received a standard diet supplemented with extra virgin oral olive oil (1 mL/100 g b.w.). For all rats, treatments with extra virgin olive oil started after 3 weeks following the ovariectomy and continued for 3 months. In addition, the baseline BMD was measured by dual-energy x-ray absorptiometry (DEXA) for the femoral bones of all rats before the OVX surgical procedure, and 2 weeks postoperatively, to confirm the induction of osteoporosis in OVX rats.

Bilateral ovariectomy was performed under complete aseptic conditions, and general anesthesia using an intraperitoneal injection of a balanced regimen combination of ketamine HCl (75 mg/kg) and xylazine HCl (10 mg/kg). A 0.5–0.1 cm single ventral midline incision, just below the first nipple of the female rat was used for bilateral ovariectomy of rats. The two ovaries were gently exteriorized from the abdominal cavity, the oviducts were ligated using a 4/0 monofilament glycomer suture (Biosyn Covidien, USA), and each ovary was excised. The abdomen was closed in two layers (muscle and skin) using the aforementioned suture in a simple pattern. Post-operatively, the operated rat was given 100 mg/kg of amoxicillin injected intramuscular (I.M) and 100 mg/kg/per os of paracetamol for four successive days. To avoid possible contamination and cannibalism, the ovariectomized rats were housed individually in a clean cage with fresh bedding for 1 week postoperatively and then re-grouped in their home cages. The skin suture can be removed after 1 week postoperatively.

### Blood and tissue sampling

At the end of the designated period of experimentation, 12 h fasted rats were euthanized under ketamine HCL (200 mg/kg) anesthesia. Blood samples from the rats were obtained in non-heparinized centrifuge tubes and then centrifuged at 3,000 rpm for 10 min to separate the sera. Each serum sample was then labeled in Eppendorf's tubes and stored at −20°C for subsequent biochemical analysis. After the collection of blood samples, the rats underwent dissection. Both the left and right femurs were promptly extracted and rinsed with a chilled saline solution. Subsequently, the left femurs were weighed and homogenized in an ice-cold saline solution utilizing a Potter-Elvehjem-type homogenizer. The resulting homogenate was then subjected to centrifugation at a force of 860 Xg for 20 min. Following centrifugation, the obtained supernatants were preserved at a temperature of −20 C for subsequent analysis. In the meantime, the right femurs were employed for the determination of bone mineral density, biomechanical analysis, and computed tomography of bone architecture. Moreover, the right femurs of all sacrificed rats were excised and cleaned of adjacent tissue, wrapped in saline-soaked gauze bandages, and stored at −20 C for computed tomography (CT) and biomechanical analysis.

### Bone mineral density

Determination of the BMD of the rat's whole femurs was conducted in a manner that ensured objectivity, utilizing the Lunar Prodigy Advance by dual-energy x-ray absorptiometry (DEXA), which was provided by GE Healthcare (Chicago, IL, USA), specifically in the small-subjects mode. The scans were performed with a pixel size of 0.125 × 0.25 mm, a line spacing of 0.0254 cm, and a point resolution of 0.0127 cm. All samples were measured three times and the mean values were calculated. BMD was represented in g/cm^2^, according to Castaneda et al. (7).

### Biochemical analysis

Serum total protein (TP) and creatinine (Cr) levels were determined calorimetrically using a colorimetric assay kit, Spinreact (Girona, Spain), Cat.No:1001290 and 1001110, respectively. Both serum and bone calcium (Ca) and phosphorus (P) levels were measured using a colorimetric assay kit, Spinreact (Girona, Spain), Cat.No:1001061, and MD1001155, respectively. The serum activity of alkaline phosphatase (ALP) was assessed using a kinetic photometric assay kit, Spinreact (Girona, Spain), Cat.No:41245, according to Yoon et al. (8).

The bone activity was determined using an enzyme-linked immunosorbent assay (ELISA) technique with the Rat bone alkaline phosphatase (BALP) ELISA kit, MyBioSource Company (California, USA), Cat.No: MBS164916, as per the manufacturer's instructions. Serum levels of both parathyroid hormone (PTH) and estradiol (E2) hormone were assessed using the Scantibodies ELISA kit (Cat.No: 3KG151), Santee, CA, USA, and the BioVendor RAT estradiol ELISA kit (Cat.No: RTC009R), respectively. Pro-inflammatory cytokines, including tumor necrosis factor-α (TNF-α) and interleukin-1β (IL-1β), were measured using commercially available ELISA kits R&D systems, Minneapolis, USA; Cat.No: 45-TNFRT-E01.1 and RayBiotech, Inc Company, Cat.No: ELR-IL1beta-001C, respectively. The bone malondialdehyde (MDA), reduced glutathione (GSH), and superoxide dismutase (SOD) levels were determined using Rat-specific ELISA kit Cat No: CELI-66086r, Cat.Nos: CSB-E12144r, CSB-E08555r, respectively (Cusabio Biotech, Wuhan, China). Catalase (CAT) was determined using an ELISA kit obtained from MyBioSource company, Cat.No: MBS2600683.

### Computed tomography analysis

The effects of olive oil treatment on the cortical and trabecular bone structures of the rat femurs for the experimental groups were detected using a multislice CT scanner (Aquilion ONE Toshiba; Toshiba America Medical Systems). The acquisition settings at a clinically typical were 120 kV, 5 mA, voxel size 6 μm, and slice thickness 0.2 mm with bone window image reconstructions. An initial scout view was performed to check for symmetry and to ensure that the entire region was included in the image. Subsequently, transverse (axial), sagittal, coronal, and oblique CT scans of each femur were reconstructed. The bone thickness (BT) of cortical dense (compact) bone in the mid-diaphyseal regions of the femur and bone tissue mineral densities (TMD) of the cortical bone and trabecular (cancellous) bone at the femur extremities were quantified using a quantitative CT method yielding workstation. The appropriate volume of the region of interest (ROI) of the femoral bone was then chosen for analysis. The 3D images were obtained for visualization and display, according to Yoon et al. (8).

### Biomechanical examination

The mechanical properties of the bone were determined by three-point bending and compression tests of the left femurs. The press head and the two support points were rounded to avoid shear load and cutting. For the three-point bending test, the femur specimen was positioned horizontally with the anterior surface upwards, centered on the supports, and the pressing force was applied vertically to the mid-shaft of the femur. For the compression test, the femur specimen was placed centrally between two parallel steel plates attached to the materials-testing device (LLOYD, Germany). The biomechanical tests were performed in a blinded fashion on a material-testing machine (LLOYD). The Load (force) was applied at a span of 20 mm, and the deformation/displacement rate was 2 mm/min until failure occurred. For each femur specimen of the treatment group, the maximum load, stiffness, maximum stress, and Young's modulus were calculated and obtained from the load-deformation curve, according to Jamsa et al. (9).

### Histopathological examination

Serial sections from the rats' femurs were fixed in 10% neutral buffer formalin for 48 h before undergoing decalcification in a 10% EDTA solution with daily exchanges for 6 weeks. The decalcified samples were then dehydrated in increasing concentrations of ethyl alcohol, cleared in xylene, and finally embedded in liquid paraffin wax. These decalcified sections from all groups were sliced at a thickness of 4 μm and stained using hematoxylin and eosin (H&E) for histopathological examinations. A histopathologist blind to the treatment group status of individual animals performed the histopathologic evaluation.

### Statistical analysis

GraphPad Prism 5.0 software (GraphPad Software Inc., San Diego, California, USA) and a statistical software package (SPSS 15.0 for Microsoft Windows, SPSS Inc.) were used to detect statistical differences among all tested parameters. All data is presented as mean ± standard deviation (SD; *n* = 6). Statistical comparisons were performed using one-way analysis of variance (ANOVA). Probability values of (*P* < 0.05) were considered statistically significant.

## Results

### Effect of olive oil on BMD and biochemical parameters

The results of the present study revealed that OVX rats exhibited a significant reduction (*P* < *0.05*) in BMD, and serum TP levels compared to control (non-treated) rats. There was a significant increase (*P* < 0.0001) in serum Cr level observed in treated rats in comparison to non-treated animals. The administration of olive oil yielded a notable increase (*P* < *0.001*) in BMD level by 90.9% compared to the OVX group. In addition, olive oil administration to the OVX group resulted in a significant increase (*P* < *0.05*) in serum TP levels by 15.6% and a significant (*P* < *0.0001*) decrease in Cr level by 19.8% in comparison with the OVX group (Table 1).

**Table 1:** Bone mineral density, serum total protein, and serum creatinine levels in all experimental groups.

**Variables**		**Control**	**Olive oil**	**OVX**	**OVX + olive oil**
BMD (g/cm^2^)	Mean ± SD	0.19 ± 0.02	0.20 ± 0.02	0.11 ± 0.03^**^	0.21 ± 0.01^##^
	%Of change		+5.3^a^	−42.1^a^	+10.5^a^; +90.9^b^
Serum TP (g/dL)	Mean ± SD	7.2 ± 0.4	7.6 ± 0.3	6.4 ± 0.4^*^	7.4 ± 0.5^#^
	%Of change		+5.6^a^	−11.1^a^	+2.8^a^; +15.6^b^
Serum Cr (mg/dL)	Mean ± SD	0.70 ± 0.05	0.66 ± 0.04	0.91 ± 0.05^***^	0.73 ± 0.02^###^
	%Of change		−5.7^a^	+30.0^a^	+4.3^a^; −19.8^b^

Data were expressed as mean ± SD, *n* = 6. OVX, ovariectomized; BMD, bone mineral density; TP, total protein; Cr, creatinine. ^*^P < 0.05, ^**^P < 0.001, and ^***^P < 0.0001 compared with the control group. ^#^*P* < 0.05, ^*##*^*P* < 0.001, and ^*###*^*P* < 0.0001 compared with OVX group. a and b percentage (%) of change compared with control and OVX groups, respectively.

**Figure 1:**
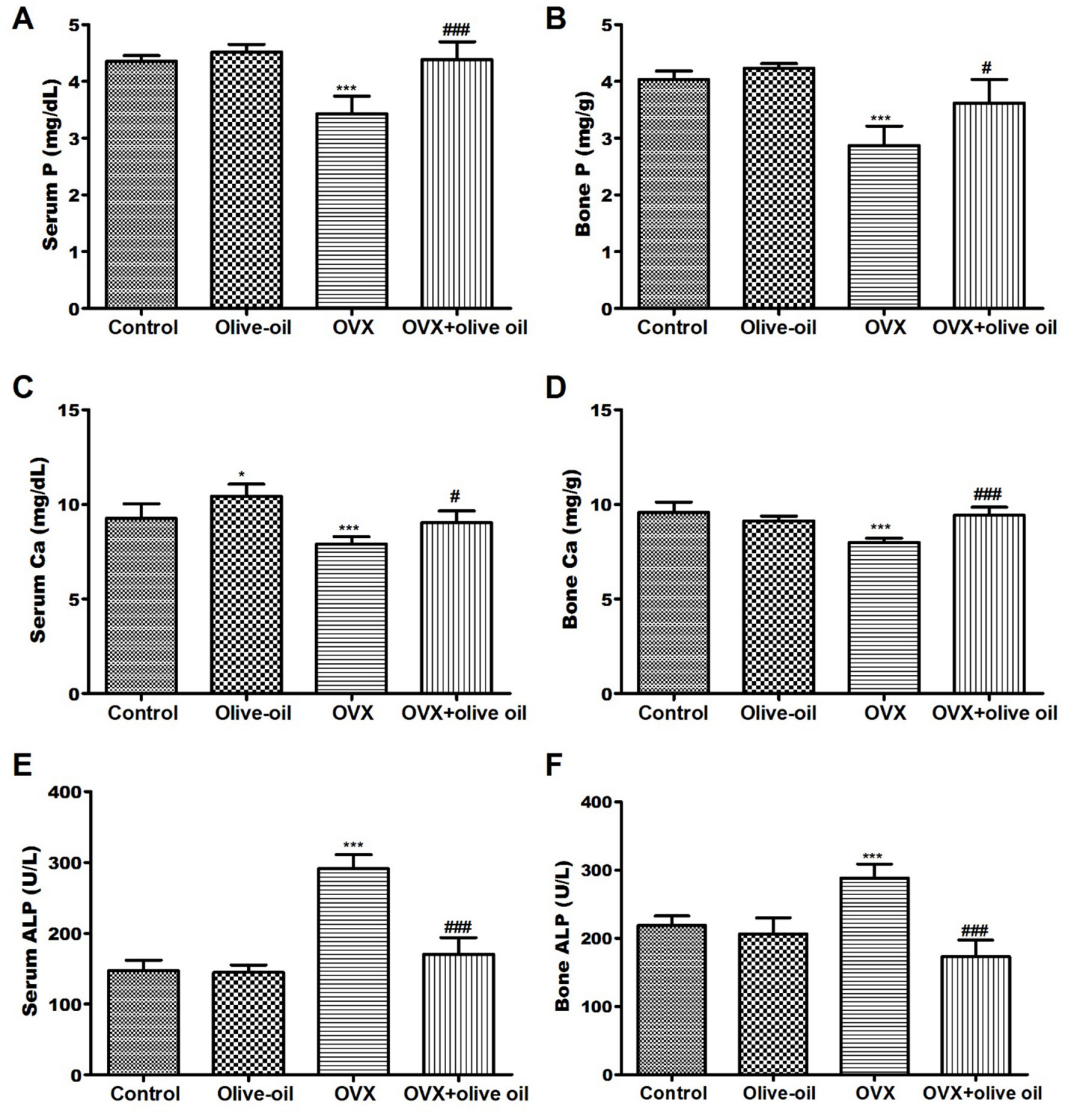
Effect of olive oil on serum BMD and biochemical parameters in all experimental groups including **(A)** serum P, **(B)** bone P, **(C)** serum Ca, **(D)** Bone Ca, **(E)** serum ALP, **(F)** bone ALP. P, phosphorous; Ca, calcium; ALP, alkaline phosphatase. *P < 0.05, and ***P < 0.0001 in comparison with control group. ^#^*P* < 0.05 and ^*###*^*P* < 0.0001 in comparison with OVX group.

The OVX group exhibited a notable reduction (*P* < 0.001) in both serum and bone levels of calcium (Ca) and phosphorus (P) compared to the control group. The OVX group also exhibited a significant (*P* < 0.001) increase in ALP activities in serum and bone. While in the OVX + olive oil group, there was a significant elevation in both bone and serum levels of Ca and P compared to the OVX group. Treatment rats with olive oil also resulted in a significant reduction (*P* < 0.001) in ALP activities by 41.6 and 39.9% in both bone and serum, respectively compared to the OVX group, as shown in Figure 1.

### Effect of olive oil on hormonal parameters

**Figure 2:**
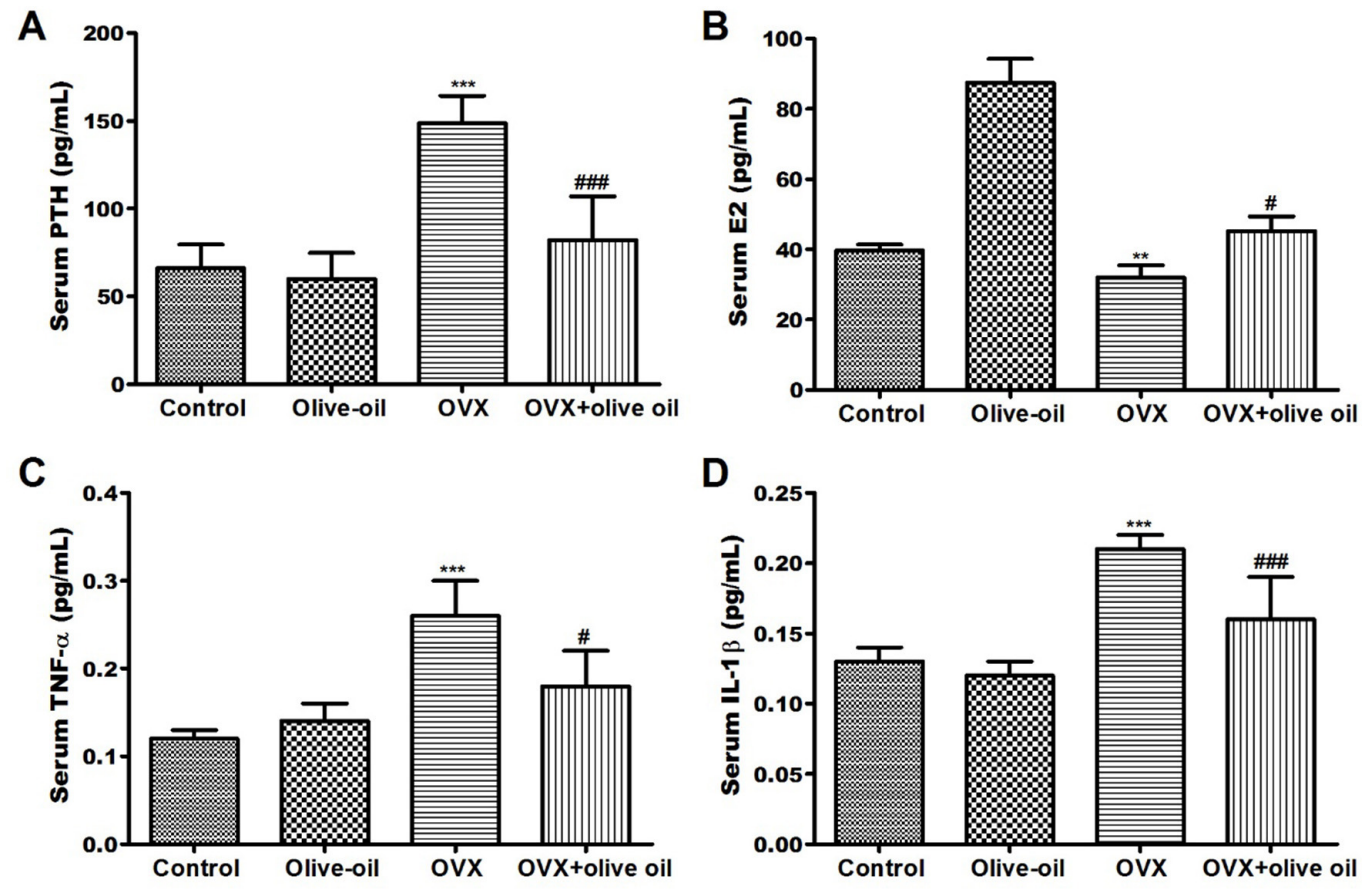
Effect of olive oil on level changes of serum hormones and bone turnover markers in all experimental groups including **(A)** parathyroid hormone (PTH), **(B)** estradiol (E2) hormone **(C)** tumor necrosis factor-α (TNF-α), and **(D)** interleukin 1 beta (IL-1β) in all experimental groups. OVX: ovariectomized. **P < 0.001 and ***P < 0.0001 in comparison with control group. ^#^*P* < 0.05 and ^*###*^*P* < 0.0001 in comparison with OVX group.

As a consequence, there was no statistically significant difference (*P* > 0.05) observed between both the olive oil group and control group in serum parathyroid hormone (PTH) levels but there was a significant elevation in estradiol (E2) hormone levels (*P* < 0.05). However, as anticipated OVX group was associated with significantly high PTH levels and reduced E2 levels compared with the control group. Contrariwise, olive oil supplementation was found to attenuate levels of such hormones. Serum PTH level was significantly decreased by 44.7%, and serum E2 level was increased by 41.1% in the OVX + olive oil group compared to the OVX group (Figures 2A, B).

### Effect of olive oil on pro-inflammatory cytokines

The TNF-α levels were significantly increased in the OVX group by 116.7% compared with the control group. Fortunately, olive-OVX rats exhibited a significant decrease (*P* < *0.05*) in TNF-α levels. As well as, a significant reduction (*P* < *0.05*) in TNF-α levels by 30.8% compared to the OVX group was observed in OVX + olive oil rats (Figures 2C, D). There was a significant increase (*P* < *0.05*) in IL-1β level in the serum of OVX rats when compared to the control group. At the same time, the OVX + olive oil rats showed a significant decrease (*P* < 0.001) in IL-1β level by 23.8% compared with the OVX group (Figures 2C, D).

### Effect of olive oil on oxidative stress and antioxidant biomarkers

There was a significant increase (*P* < *0.001*) in malondialdehyde (MDA) levels in the OVX rats compared to the control group. However, in the OVX + olive oil group, the levels of bone MDA levels were significantly reduced (*P* < 0.0001) compared with the OVX group (Table 2). For glutathione (GSH), there was a significant decrease (*P* < 0.001) in the OVX group in comparison with the control group. Whereas, the OVX + olive oil group exhibited a significant elevation (*P* < 0.05) in GSH content to the OVX rats by 81.3%. The superoxide dismutase (SOD) activity, in the OVX group showed a significant decrease (*P* < 0.001) in SOD activities compared to the control group. Olive oil treatment was found to enhance the activity of this enzyme in the OVX + olive oil group compared with the OVX group (*P* < 0.001). Regarding catalase (CAT) bone activity, there was a significant difference (*P* < 0.05) between both the olive oil and control groups. On the contrary, like other antioxidant enzymes, OVX rats were associated with a significant decrease (*P* < 0.001) in CAT activities by 46.2% relative to the control group. Whereas, the OVX + olive oil group increased CAT activity by 14.3% compared to the OVX group (Table 2).

**Table 2:** Oxidative stress and antioxidant biomarkers in all experimental groups.

**Variables**		**Control**	**Olive oil**	**OVX**	**OVX + olive oil**
Bone MDA (nmoL/mL)	Mean ± SD	0.17 ± 0.01	0.17 ± 0.02	0.34 ± 0.08^***^	0.19 ± 0.02^###^
	%Of change		0.0^a^	+100.0^a^	+11.8^a^;−44.1^b^
Bone GSH (ng/mL)	Mean ± SD	0.28 ± 0.04	0.48 ± 0.03	0.16 ± 0.05^***^	0.29 ± 0.03^###^
	%Of change		+71.4^a^	−42.9^a^	+3.6^a^; +81.3^b^
Bone SOD (U/mL)	Mean ± SD	0.43 ± 0.03	0.45 ± 0.03	0.19 ± 0.03^***^	0.30 ± 0.01^###^
	%Of change		+4.7^a^	−55.8^a^	−30.2^a^; +57.9^b^
Bone CAT (ng/mL)	Mean ± SD	0.26 ± 0.04	0.36 ± 0.08^*^	0.14 ± 0.04^**^	0.16 ± 0.01
	%Of change		+38.5^a^	−46.2^a^	−38.5^a^; +14.3^b^

Data were expressed as mean ± SD, *n* = 6. OVX, ovariectomized; MDA, malondialdehyde; GSH, reduced glutathione; SOD, superoxide dismutase; CAT, catalase. ^*^P < 0.05, ^**^P < 0.001, and ^***^P < 0.0001 in comparison with control group. ^*###*^*P* < 0.0001 in contrast with the OVX group. a and b percentage (%) of change compared to control and OVX groups.

### Effect of olive oil on bone structure

**Figure 3:**
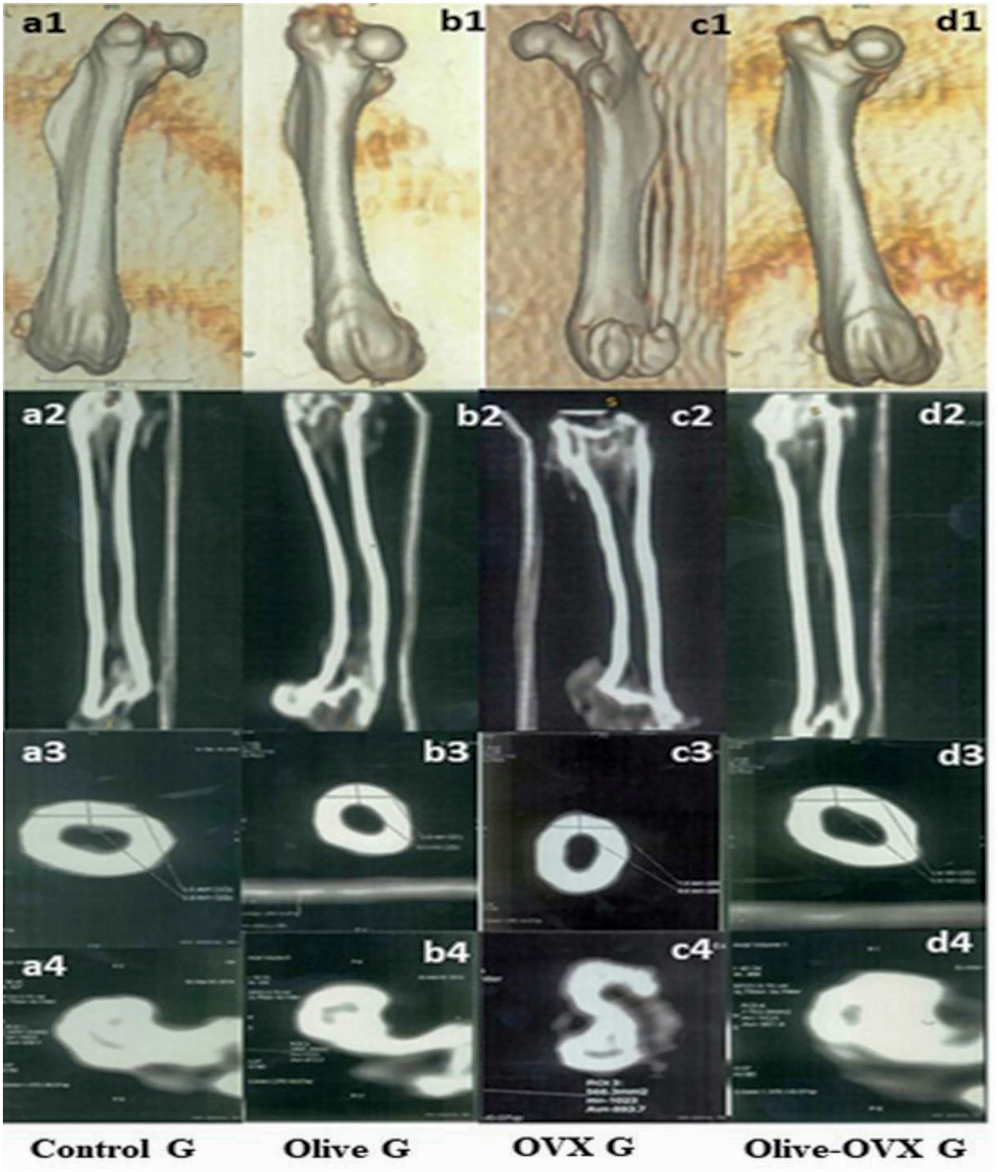
CT scan of the rat femurs in all experimental groups. a1, b1, c1, d1 represent the 3D images of the femur within the rat group. a2, b2, c2, d2 Coronal scan for the femur within the rat group. a3, b3, c3, d3 axial scan for the cortical BT of the femur within the rat group. a4, b4, c4, d4 axial scan for the trabecular bone TMD of the femur within the rat group.

The CT analysis of the structural parameters of the rat femurs showed significant improvements in the trabecular and cortical bone thickness (BT) and bone tissue mineral density (TMD) in the OVX + olive oil group (*P* < *0.05*) compared to the OVX group. There was no significant difference between the olive and control groups in the femoral structural parameters (*p* < *0.06*). The descriptive details of the rat femur structural parameters are shown in Table 3 and Figure 3.

**Table 3:** CT analysis of the rat femurs' trabecular and cortical parameters.

**Group**	**BT (mm)**	**TMD (mg/cm^3^)**
Group I (control)	0.87 ± 0.06^a^	1701.23 ± 89.37^a^
Group II (olive oil)	0.79 ± 0.05^a^	1601.12 ± 85.28^a^
Group III (OVX)	0.5 ± 0.2^b^	1170.30 ± 91.03^b^
Group IV (olive-OVX)	0.81 ± 0.05^a^	1657.36 ± 90.63^a^

BT, bone thickness; TMD, Tissue mineral density. ^a,b^Medians with different superscript letters in the same column are significantly different at *P* < 0.05. All results are expressed as Mean ± SD.

### Effect of olive oil on biomechanical properties of the femur

The results of the femur biomechanical tests indicated that the OVX + olive oil group had a higher maximum load and stiffness (*P* < *0.05*) and showed significant increases in Young's modulus and maximum stress (*P* < *0.01*) when compared to the OVX group. The olive oil group showed no significant differences compared with the control group. However, the OVX group significantly reduced biomechanical parameters compared to the control group (Table 4).

**Table 4:** The estimated biomechanical parameters of the rats' femur.

**Group**	**Load (N)**	**Stiffness (N/mm)**	**Stress (Mpa)**	**Young's modulus (Mpa)**
Group I (control)	159.37 ± 6.03^a^	278.13 ± 10.46^a^	147.12 ± 8.25^a^	8219.75 ± 337.60^a^
Group II (olive oil)	153.11 ± 5.53^a^	266.75 ± 8. 49^a^	141.39 ± 7.91^a^	8193.36 ± 305.68^a^
Group III (OVX)	131.47 ± 4.27^b^	217.62 ± 19.35^b^	102.64 ± 9.17^b^	5846.41 ± 139.72^b^
Group IV (olive-OVX)	155.84 ± 5.51^a^	263.98 ± 13.21^a^	143.56 ± 8.04^a^	8184.09 ± 285.49^a^

^a,b^Medians with different superscript letters in the same column are significantly different at *P* < 0.05. All results are expressed as Mean ± SD.

### Effect of olive oil on histopathological architecture

**Figure 4:**
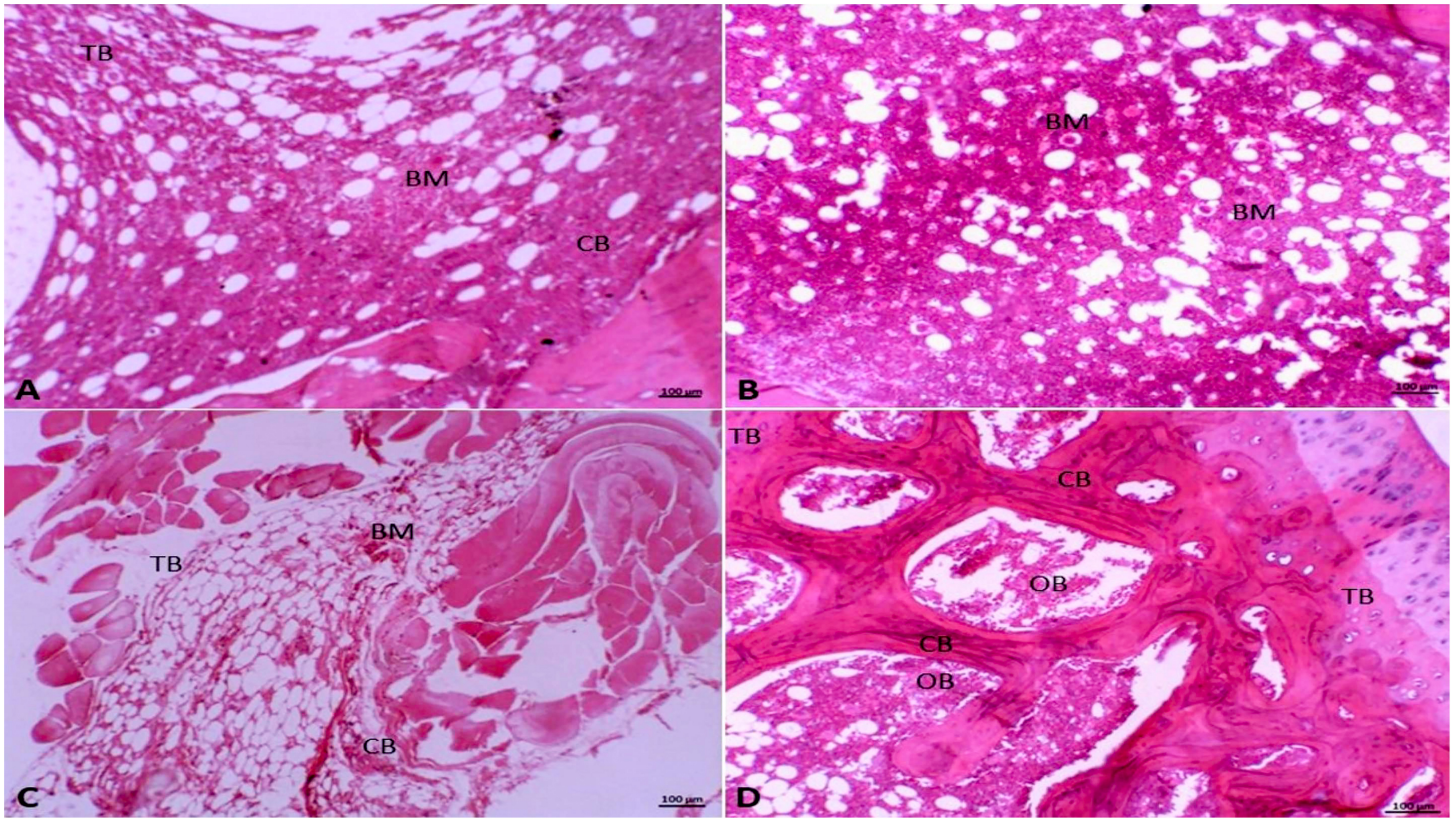
Photomicrograph of the femur bone tissue stained with hematoxylin and eosin (HE, 10x). **(A)** Showing normal cortical (CB) and trabecular bone (TB), in addition to normal bone marrow (BM) in the Group I (control group). **(B)** In Group II (olive-oil group) normal bone marrow stem cells (BM) appear as small cells with large basophilic nuclei and scanty cytoplasm with scattered fat globules. **(C)** Showing histological alteration of trabeculae (TB) and cortical bone (CB) with bone marrow (BM) degeneration and widening of bone marrow spaces in Group III (OVX-group). **(D)** In Group IV (olive-OVX group) showing normal cortical bone (CB) with normal osteoblasts (OB) and normal osteoid matrix with normal bony trabeculae (TB).

The Photomicrograph of the rat femoral bone sections stained with H&E revealed that the control group exhibited regular cortical and trabecular bone architectures. Additionally, the bone marrow was characterized as of normal cellularity, and normal bone marrow spaces were seen between the trabeculae (Figure 4A). Furthermore, sections in the olive group were nearly similar to the control group. It was manifested by normal bone marrow stem cells that appeared as small cells with large basophilic nuclei and scanty cytoplasm with scattered fat globules (Figure 4B). However, in the OVX rats, the stained sections exhibited a clear histological modification of both trabeculae and cortical bone with the widening of bone marrow spaces. These alterations appeared in bone marrow degeneration and distortion of the endosteal surface, which displayed erosion without osteoblast and osteogenic lining (Figure 4C). Interestingly, the OVX + olive oil group revealed marked improvement in the histological architecture compared to the OVX group. There was a significant improvement in the cancellous and cortical bone appearance with less widened bone marrow spaces. The osteocytes were distributed within the bone matrix, and the endosteal surface appeared smooth and lined osteoblast cells (Figure 4D).

## Discussion

Numerous studies have presented evidence of bone loss stimulated by insufficient levels of estrogen in rats that underwent ovariectomy (8, 10). Furthermore, other investigators have reported an average femoral BMD loss of 15 and 20% post ovariectomy (5). Our findings revealed that the utilization of olive oil resulted in a significant 90.9% increase in BMD compared with the OVX group. This is consistent with the findings of Liua et al. (11), Saleh and Saleh (12), and Hagiwara et al. (13) who observed olive oil supplementation can effectively mitigate trabecular bone loss and promote an increase in BMD in the lumbar spine and left femur of OVX rats. This favorable outcome could be attributed to the phenolic compounds within olive oil, which exhibit antioxidant properties by scavenging free radicals (12, 13). As a result, bone cells are shielded from oxidative harm, thereby contributing to the observed effect (14).

It is worth noting that osteoporosis can cause changes in blood and bone parameters. Cr is considered one of the most notable indicators of bone imbalance and is used for monitoring purposes (12). In this study, the levels of serum Cr were found to be significantly elevated in cases of ovariectomy-induced osteoporosis, which aligns with previously reported findings (15, 16). On the other hand, when olive oil supplementation was in the OVX + olive oil group, there was a significant decrease in Cr levels. Likewise, Hassan et al. (17) noted that the consumption of oleuropein appeared to have a significant attenuating effect on Cr levels compared to OVX-rats.

Menopause and ovariectomy are associated with imbalances in the rate of absorption and reabsorption of Ca and P by the small intestine and kidneys. Consequently, alterations in estradiol levels resulted in a notable reduction in blood Ca and P concentrations (5, 13). Our findings support this perspective, as OVX-rats showed a significant decrease in both Ca and P levels in the serum and bones (*P* < 0.0001). While there was a notable increase (*P* < *0.0001*) in the levels of Ca and P with the application of olive oil treatment. Similarly, various studies have shown that olive oil effectively prevents hypocalcemia caused by ovariectomy in OVX + olive rats (12, 14). This could be explained by the presence of phenols in olive oil such as hydroxytyrosol and oleuropein that enable the deposition of Ca ions in osteoblastic cells and hinder the formation of osteoclasts. Additionally, olive oil aids in the absorption of Ca in the intestines and acts as a significant reservoir of gamma-linolenic acid, which reduces the elimination of Ca, decreases bone reabsorption, and affects bone turnover markers (18).

There was a correlation between estrogen deficiency and expedited bone remodeling, wherein bone resorption surpassed bone formation. ALP is acknowledged as a factor that determines the process of osteoblastic differentiation (13, 19). The activation of ALP prompts the mineralization of matrix proteins through the hydrolysis of pyrophosphate and inorganic phosphate (16, 20). In this study, the OVX group demonstrated a significant increase in ALP activities in serum and bone compared to the control group, indicating heightened osteoblastic activity and enhanced bone formation.

Our findings indicate that the inclusion of olive oil in the diet of rats that have undergone ovariectomy can protect against osteoporosis by regulating the levels of PTH and E2 hormones. These results contradict the study conducted by Yoon et al. (8) that showed a significant decrease in serum estradiol levels in the OVX group compared to the sham group. The elucidation for this enhancement in bone structure may be related to the heightened concentrations of E2 and the abundance of mono-unsaturated fatty acids that are found in olive oil (21). The consumption of extra virgin olive oil polyphenols regulates the expression of genes associated with estrogen response in the uterus in a manner that mimics the effects of E2 (21). Furthermore, olive oil may contain phytoestrogens, similar to those found in other plants or their extracts, which could potentially have milder effects similar to estradiol-17β benzoate and contribute to the preservation of bone mass during the post-menopausal period by acting as osteoprotective agents (22).

The immune system assumes a pivotal function in the pathophysiology of osteoporosis after menopause. Consequently, an elevation in pro-inflammatory cytokines such as TNF-α and IL-1β can worsen osteoporosis as they participate in bone turnover and act as potent stimulants of bone resorption (15, 23). It has been demonstrated that estrogen deficiency resulting from ovariectomy leads to the production of IL-1β and TNF-α by osteoblasts, which in turn activate and differentiate osteoclasts, ultimately increasing bone resorption (10, 21). Our results for the significantly high IL-1β and TNF-α levels observed in the OVX rats could suggest that olive oil may have an anti-inflammatory effect in OVX-induced osteoporosis.

There is strong evidence that the architecture of cortical and cancellous bone plays a significant role in bone strength and determines its biomechanical properties. The CT measurements of bone structures have been proven much earlier sensitive during the progress of the bone morphopatholgical changes, which may be considered a predictive tool for the diagnosis of changes in bone architecture (8). This methodology might be further extended to study the effects of bone morphology on its dynamic behavior and resistance to fracture. Therefore, it seems that both BT and TMD measurements are in agreement with the serum measurements of bone biochemical markers. Our result showed significant improvements in BT and TMD in the trabecular and cortical bone in the olive + OVX group (*P* < *0.05*) compared to the OVX group. These results indicate an enhancement of osteoblast activity and their maturation, synthesis of organic bone matrix components, and bone mineralization. Similar findings were previously reported (8, 24).

Changes in mechanical properties of bone with olive oil treatment is a dispute between previous studies. While some studies (5, 13) have shown that olive oil creates a bone protective effect by increasing bone formation, other studies (25, 26) have implied a statin effect by preventing resorption rather than increasing bone formation. The most important marker of osteoporosis is the severe decrease in bone strength, which reflects the decrease in the numbers and functions of osteoblasts and directly results in bone fractures. Measurement of cortical and trabecular bone biomechanics is a useful correlate of bone density either by the three-point bending test and/or compression test (9). In this study, the result of the biomechanical strength of the rat femur revealed a significant difference in load (155.84 ± 5.51), stiffness (263.98 ± 13.21), stress (143.56 ± 8.04), and Young's modulus (8184.09 ± 285.49) in the olive + OVX group when compared with the OVX group. While no differences were found between the olive + OVX group and the control group in the aforementioned parameters. Our results demonstrate that olive oil prevented the decrease in bone mineral density and enhanced bone strength after ovariectomy. These findings were in agreement with Castaneda et al. (7) and Melguizo-Rodríguez et al. (20).

Trabecular and cortical bone microarchitecture is considered to be a proper predictor of OVX-induced bone loss and bone structure deterioration (11). Bone thickness (BT) and bone tissue mineral density (TMD) are key measures characterizing the 3D structure of trabecular and cortical bone. Thus, in the present study, we examined these parameters using CT scanning in OVX rats after the administration of olive oil. The CT analysis of the structural parameters of the rat femurs showed significant improvements in the trabecular and cortical bone thickness (BT) and bone tissue mineral density (TMD) in the OVX + olive oil group (*P* < *0.05*) compared to the OVX group that showed dramatic deterioration of the normal trabecular and cortical bone micro-architecture after ovariectomy. These findings were consistent with the results obtained by Castaneda et al. (7) and Yoon et al. (8). Taken together, these results suggested that olive oil might ameliorate trabecular micro-architecture and represent a good prevention and treatment method for estrogen-deficient osteoporosis.

In the present study, the histological alterations of the trabecular and cortical bone in the OVX group were restored by olive oil administration in the olive + OVX group. The enhancement of osteoblast activity and their maturation in the OVX + olive group was the reason for the significant increase in collagen density, synthesis of organic bone matrix components, and bone mineralization in the femur of treated rats. While sections in the Olive group were nearly similar to the control group. These results were quite similar to findings obtained by Saleh and Saleh (12), Hagiwara et al. (13), and Melguizo-Rodríguez et al. (20). These results indicated that olive oil administration can significantly improve the structural parameters of both cortical and trabecular bone density in osteoporotic rats.

Although this study indicated that dietary use of olive oil effectively mitigates the effects of ovariectomy-induced osteoporosis in rats, there are some limitations of this study. First, a full exploration of the olive oil extraosseous effects on tissues is required, particularly hepatic, cardiac, and renal tissues. Second, to improve therapeutic impact of olive oil supplementation, dose and treatment length must also be investigated further. Third, molecular analysis of genes or proteins involved in bone formation (e.g., Runx2, ALP) and resorption (e.g., RANKL, OPG) to provide mechanistic insights into olive oil effects. Fourth, future analysis to other markers, such as osteocalcin or tartrate-resistant acid phosphatase (TRAP), to provide a more comprehensive analysis is important. Furthermore, further deep studies are required to provide more granular data on trabecular spacing, connectivity, and other microarchitectural parameters to enhance understanding of bone quality improvements after the treatment with olive oil.

In conclusion, olive oil dietary intake is effective in reducing the impact of osteoporosis induced by ovariectomy in rats, suggesting a potentially feasible treatment option for postmenopausal osteoporosis that has a beneficial effect on bone architecture without any detrimental side effects on women's health. Further clinical studies are required to validate the utilization of olive supplements in individuals with osteoporosis and ascertain the underlying mechanism of its impact.

## Data Availability

The datasets presented in this study can be found in online repositories. The names of the repository/repositories and accession number(s) can be found in the article/supplementary material.
